# Promoting gender, equity, human rights and ethnic equality in neglected tropical disease programmes

**DOI:** 10.1093/trstmh/traa159

**Published:** 2021-01-13

**Authors:** P S Mbabazi, S Del Pino, C Ducker, L Dean, H Broekkamp, W Prasetyanti, J Jacobson, A Krentel, M Seunik, A L Bustinduy, M Malecela

**Affiliations:** World Health Organization, Department of Control of Neglected Tropical Diseases, Geneva, Switzerland; Pan American Health Organization/World Health Organization Regional Office for the Americas, Washington, DC, USA; Tro Da Global Health Consulting, UK; Liverpool School of Tropical Medicine, Liverpool, UK; NLR, the Netherlands; NLR, Indonesia; Managing Partner, Bridges to Development, USA; Bruyère Research Institute, University of Ottawa, Canada; Grand Challenges Canada, Canada; London School of Hygiene &Tropical Medicine, London, UK; World Health Organization, Department of Control of Neglected Tropical Diseases, Geneva, Switzerland

**Keywords:** inequalities, gender, equity, human rights, ethinic, neglected tropical diseases

## Abstract

Limited attention to tackling neglected tropical diseases (NTDs) through the lenses of gender, equity, ethnicity and human rights inadvertently undermines progress due to the exclusion of subgroups in populations living in conditions of vulnerability. Supporting national NTD programmes to make equity analysis part of their routine activities and revitalising intersectoral collaboration will be essential to achieve effective, sustainable service delivery with a person-centred approach. Gender, equity, human rights and ethnic equality for NTD programmes should therefore be incorporated in multisectoral engagements.

The 2030 Agenda for Sustainable Development emphasises the importance of ensuring equitable health outcomes and healthcare delivery. Transformative in its gender-sensitive approach and vision for equity in its quest to leave no one behind, the Agenda is universal in urging all countries to make progress towards implementing the ideals of human rights and inclusiveness. Yet so far, multisectoral approaches to tackling neglected tropical diseases (NTDs) have had little more than an episodic focus on gender, equity and ethnic equality, often being considered from a biomedical standpoint, with a cursory exploration of the complex sociocultural factors that influence patients’ experience, ranging from susceptibility to living with life-altering morbidity. Individuals living in vulnerable situations, such as migrant workers, refugees, indigenous peoples and those with physical and mental disabilities, are often more susceptible to NTD-associated infection and morbidity, heightening their potential social exclusion. If the goals outlined in the WHO 2021–2030 NTD road map are to be achieved,^1^ it is vital that those living in situations of vulnerability have the same opportunities to receive NTD treatments as those living in countries where elimination has already occurred.

Despite the impact of traditional gender roles, little attention is given to gendered NTD inequalities. Some NTD programmes have invested in considering gender, but the work is patchy without a holistic view across all NTDs or even within a specific disease. There are examples of both women and men being left behind in NTD programmes. For example, women living with leprosy tend to receive treatment later than men because of societal and internalised stigmatising attitudes, and the gender insensitivity of leprosy services. These factors also mean that a proportion of women with leprosy have yet to be identified at all.^2^ Gendered analysis of NTD outcomes for individuals tends to focus on disease outcomes predominantly for women, with limited exploration of the illness experience of men and people who are non-binary individuals.^3^ The biological consequences of NTD infections such as trachoma, schistosomiasis and leishmaniasis are better understood for women. However, we must acknowledge that the disabling aspects of some diseases—such as female genital schistosomiasis, which affects women's sexual and reproductive health—are gender-specific and known to be neglected within disease-specific control strategies.^4^ Other studies also show that, compared with women, men are less likely to access community-based treatments for lymphatic filariasis, suggesting that there is a need for gender-sensitive analyses of men's lives and masculinities in relation to NTDs. Indeed, masculinity is generally associated with higher health disparities, with men often preferring to face risk and physical discomfort rather than be associated with stereotypically ‘feminine’ traits.^5^ Furthermore, despite some reflections on the impact of gender on health outcomes for some NTDs, little research has explored how this influences comorbidities such as mental health, quality of life and experience through time. Intersectional research exploring how gender interacts with other axes of inequality, such as age and education and with broader contextual factors such as conflict and poverty, to shape the NTD experience, is also limited.

Achieving universal access to interventions against NTDs calls for new working methods to reach urban populations, conflict areas, ethnic minorities and migratory communities.^6^ These include new approaches to ensure that newly recognised NTDs such as snakebite envenoming are not left behind due to old ways of programming,^7^ and are supported by new forms of data collection and analysis to identify inequities in health and evaluate policies and programmes. Current NTD information systems do not sufficiently capture parameters for health equity influenced by higher levels of poverty, illiteracy and lack of access to basic services such as water and sanitation. Nor is there information on ethnicity collected to understand how different ethnic groups are affected. These evidence gaps have stymied efforts to move beyond simply describing disease burdens to tackling the larger social and structural processes that influence vulnerability across the NTD continuum from exposure to long-term care.

Programmes based on human rights have been shown to improve service delivery and enhance equality, equity, inclusiveness and accountability. Yet for NTDs, formal mechanisms of encouraging accountability for inclusion and human rights in NTD programming are rare, despite initial efforts in the early 2000s.^8^ Advocacy promoting NTDs as a ‘best buy’ in global health can hide the larger costs associated with interventions to tackle the wider determinants of disease. Importantly, many of these hidden costs may also be borne by the NTD volunteer workforce, adding a biased burden to communities. Limited acknowledgement of the full cost of tackling NTDs weakens the transformative potential of NTD programming.

The NTD 2021–2030 road map presents a paradigm shift, which calls for increased coimplementation, better integration with the wider health system and more holistic approaches to the management of NTDs. Without better understanding of the illness experience associated with NTDs and how this varies by axes of inequality, and between different NTDs, achieving and sustaining the gains from such a paradigm shift will be difficult. The epidemiology of NTDs offers pragmatic opportunities to test these shifts, which should be measured not simply through whether gender-disaggregated data are being collected and reported, but by the extent to which programmatic decision-making authority and implementation are ensuring equitable delivery of NTD services.

Recent approval of seminal policies at the ministry level^9^ and creation of a new WHO Technical Working Group on gender, equity and human rights for NTDs^10^ underscore calls to better understand determinants of health inequities associated with NTDs. This new momentum should be sustained to support equitable action against NTDs. Revitalising intersectoral engagement to support equity analyses for national NTD programmes will be essential. Such joint efforts are needed to attain well-being for individuals affected by NTDs, both as local and global citizens.

## Data Availability

None.
